# Combined linaclotide and polyethylene glycol electrolyte for colonoscopy preparation: a network meta-analysis of 14 randomized controlled trials

**DOI:** 10.1007/s00384-025-04931-9

**Published:** 2025-06-18

**Authors:** Mohamed S. Elgendy, Islam Rajab, Qasi Najah, Mohamed A. Faheem, Omar K. Elsawy, Hosam I. Taha, Mariam Elewidi, Abdalhakim Shubietah, Dhruv Patel, Alisa Farokhian, Mohamed Abuelazm, Walid Baddoura

**Affiliations:** 1 Faculty of Medicine, Tanta University, Tanta, Gharbia Egypt; 2 Internal Medicine Department, St Joseph University Medical Center, Paterson, NJ USA; 3 Faculty of Medicine, Al Margeb University, Al Khums, Murqub, Libya; 4 Internal Medicine Department, Advocate Illinois Masonic Medical Center, Chicago, IL USA; 5 Gastroenterology and Hepatology, St. Joseph’s University Medical Center, Paterson, NJ USA

**Keywords:** Colonoscopy, Colorectal cancer screening, Polyp detection, Endoscopic bowel cleansing, Meta-analysis

## Abstract

**Purpose:**

Recent evidence supports linaclotide (Lin) for colonoscopy preparation. This network meta-analysis evaluates the combination of different pill numbers of Lin with polyethylene glycol (PEG) (high and low volumes in liters (L)) for bowel cleansing.

**Methods:**

This systematic review and frequentist network meta-analysis, conducted in October 2024, assessed randomized controlled trials (RCTs) from Scopus, PubMed, Cochrane, WOS, and Embase. Risk ratios (RR) and mean differences (MD) with 95% confidence intervals (CI) were calculated for categorical and continuous outcomes. PROSPERO ID: CRD42024618272.

**Results:**

Fourteen RCTs with 4,764 participants showed that total Boston Bowel Preparation Scale improved significantly with 2L PEG + 2Lin (MD = 2.03, 95%CI: [0.30:3.76], *P* = 0.0217), 3L-PEG + 3Lin (MD = 1.30, 95%CI: [0.42:2.18], *P* = 0.0038), and 4L-PEG (MD = 1.11, 95%CI: 0.23–1.98, *P* = 0.0129). Adenoma detection was highest with 3L-PEG + 3Lin (RR = 1.60, 95%CI: [1.05:2.43], *P* = 0.0280), while polyp detection improved with 2L PEG + 3Lin (RR = 1.72, 95%CI: [1.13:2.62], *P* = 0.0114) and 3L-PEG + 3Lin (RR = 1.33, 95%CI: [1.00:1.77], *P* = 0.0505). Procedure times were significantly reduced with 3L-PEG + 3Lin (MD = -4.6, 95%CI: [-6.24:-3.24], *P* < 0.0001), 3L-PEG + 1Lin (*P* = 0.035), and 4L-PEG (*P* < 0.01). Abdominal pain and abdominal bloating decreased with 2L PEG + 1Lin (*P* < 0.01) and 2L PEG + 2Lin (*P* = 0.021) but increased with 4L-PEG (*P* = 0.0178).

**Conclusions:**

Combining PEG with Lin improves bowel cleanliness compared to 3L-PEG, with 2L PEG + 2Lin being the most effective and well-tolerated. Despite some heterogeneity, the findings suggest that adding Lin may enhance bowel preparation with comparable safety, warranting consideration of individual patient factors.

**Supplementary Information:**

The online version contains supplementary material available at 10.1007/s00384-025-04931-9.

## Introduction

Colorectal cancer represents a significant global public health issue. It is the second leading cause of cancer-related deaths and ranks as the third most commonly diagnosed cancer worldwide [1, 2]. As the primary screening method, colonoscopy reduces colorectal cancer mortality by 40–60% [3]. However, the efficacy of colonoscopy is highly dependent on the quality of bowel preparation. Inadequate bowel cleansing can obscure mucosal visibility, leading to missed diagnoses​​. Notably, poor preparation triples the risk of missing adenomas ≥ 5 mm [4] and contributes to failed cecal intubation with increased procedure time, missed diagnoses, costs, and complication risks [5, 6].

Clinical guidelines and previous studies recommend a split-dose regimen of 4 L of polyethylene glycol (PEG) as the gold standard for bowel preparation [7, 8], and a modified split-dose regimen of 3 L is used in China, as it ensures high-quality bowel cleansing. However, patients often poorly tolerate traditional high-volume regimens (e.g., 3–4 L of PEG) due to the large fluid intake required and its unpleasant taste [9]. A low dose regime is recommended for patients at low risk for inadequate bowel preparation, according to current guidelines [10]. The combination of linaclotide (Lin) or other adjuncts may enhance bowel preparation in patients with risk factors for inadequate bowel preparation (such as functional constipation) [11]. Adverse events (AEs) such as nausea and vomiting further contribute to poor patient acceptance and compliance. This has led to increasing interest in adjunctive agents, such as Lin, to improve the efficacy and tolerability of bowel preparation.

Lin is a guanylate cyclase C agonist that enhances intestinal fluid secretion, speeds up colonic transit, and reduces visceral pain via intracellular or extracellular cyclic guanosine pathways [12]. It is used to treat chronic constipation and irritable bowel syndrome with constipation, exhibiting a strong safety profile and minimal side effects in long-term treatment [13]. Growing evidence supports the combination of PEG and Lin as an effective bowel preparation regimen. Several trials have evaluated different PEG volumes (e.g., 1L, 2L, and 3L) combined with varying doses and timings of Lin administration, highlighting improvements in bowel cleansing quality, patient compliance, and AEs profiles. [14–27] However, variability in study interventions and outcomes highlights the need for further research to determine the optimal combination protocol.

Therefore, we conducted this meta-analysis to compare the safety and efficacy of Lin in different doses combined with PEG regimens versus PEG-only regimens for colonoscopy bowel preparation. Our objective is to evaluate whether Lin-based regimens offer improved outcomes in bowel cleansing while maintaining safety and tolerability.

## Methodology

### Protocol registration

We prospectively registered this network meta-analysis (NMA) in the International Prospective Register of Systematic Reviews (PROSPERO) under the ID: CRD42024618272. The analysis was conducted in compliance with the PRISMA and PRISMA-NMA (Supplementary Table 1) statement guidelines for systematic reviews and meta-analyses [28, 29] and the recommendations outlined in the Cochrane Handbook for Systematic Reviews and Meta-Analyses [30].

### Data sources and search strategy

Two authors (M.S.E. and I.R.) independently performed a comprehensive literature search on October 15th, 2024, using the strategy outlined in Supplementary Table 2. The search was conducted across five databases, including PubMed, Web of Science, Cochrane (CENTRAL), Scopus, and EMBASE, with no language restrictions applied. Foreign language articles were translated as needed. The search strategy utilized a combination of free-text keywords and Medical Subject Heading (MeSH) terms for"Linaclotide"AND"Polyethylene glycol."Additionally, M.S.E. and I.R. conducted reference screening and a random search on Google Scholar and ResearchGate to ensure that all relevant articles were included.

### Eligibility criteria

We included randomized controlled trials (RCTs) based on the following PICO criteria:

1. Population (P): adult patients (≥ 18 years) scheduled for colonoscopy without restrictions on sex, ethnicity, or clinical setting.
2. Intervention (I): bowel preparation regimens utilizing PEG in different volumes combined with varying doses of Lin, administered in 290 µg for each dose.
3. Comparison (C): standard bowel preparation regimen using PEG alone.
4. Outcomes (O): the primary outcomes included adequate bowel preparation (success rate) and the total Boston Bowel Preparation Scale (BBPS) score. Secondary outcomes encompassed efficiency metrics such as total Ottawa Bowel Preparation Scale (OBPS) score, fluid volume required, polyp detection rate (PDR), adenoma detection rate (ADR), colonoscope insertion time, withdrawal time, total procedure time, and time to first defecation. Safety metrics included specific AEs such as abdominal pain, abdominal distension/bloating, nausea, and vomiting. Compliance metrics included the willingness to repeat the preparation.

The exclusion criteria included non-randomized trials, single-arm studies, observational studies, animal studies, reviews, incomplete data, and non-primary research like conference abstracts, protocols, letters, and case reports or series.

### Study selection

The review screening process was conducted using predefined inclusion and exclusion criteria on the Covidence software. After removing duplicates, three independent reviewers (M.S.E., M.A.F., and O.K.E.) screened titles and abstracts of the remaining records. Studies meeting the inclusion criteria underwent full-text screening, with discrepancies resolved through consultation with I.R..

### Data extraction

Five reviewers (M.S.E., M.A.F., H.I.T., O.K.E., and A.S.) independently extracted the data using a pre-designed sheet initially formatted through a pilot data extraction process on Excel (Microsoft, USA) by M.S.E. The extracted data was organized into three key sections: [1] summary characteristics of the included trials: (study ID, study design, country, total Samples, study Arms, regimen, inclusion criteria, and primary outcome). [2] baseline characteristics of patients: (demographic data including age, sex, body mass index). [3] outcomes data: (total BBPS score, successful bowel preparation, total OBPS score, fluid volume required, PDR, ADR, colonoscope insertion time, withdrawal time, total procedure time, time to first defecation, abdominal pain, abdominal distension/bloating, nausea, vomiting, composite of nausea and vomiting, and willingness to repeat the preparation). In Xinlei et al. (2024) [22], we combined Groups 2 and 3, as both regimens involved administering a single dose (290 µ.g) of Lin and a total of 2 L of PEG solution, albeit with slight variations in administration timing, allowing for a more unified data analysis in our review.

### Quality assessment

We assigned five investigators (M.E., M.S.E., M.A.F., O.K.E. and H.I.T), with at least two evaluating each paper, to independently assess the risk of bias in eligible studies using the Cochrane Risk of Bias (RoB) two [31] assessment tools. Each study was classified as having a low, some concerns, or high risk of bias based on how well the primary outcomes aligned with the following criteria: random sequence generation, allocation concealment, blinding of participants and personnel, blinding of outcome assessment, completeness of outcome data, selective reporting, and other potential sources of bias. Any discrepancies were resolved through consultation with a sixth investigator (I.R.).

### Statistical analysis

A network meta-analysis was conducted using a frequentist hierarchical model to estimate the comparative efficacy of interventions. Risk ratios (RR) were calculated for categorical outcomes, and mean differences (MD) for continuous outcomes. The analysis utilized the"netmeta"package (v 2.9.0) in R statistical software (v 4.4.2). Consistency between direct and indirect evidence was assessed to ensure model validity. Treatment rankings were determined by calculating the Surface Under the Cumulative Ranking curve (SUCRA), and results were presented as relative rankings and probability distributions for each outcome. Higher SUCRA values indicate better primary and secondary outcomes and a lower incidence of AEs. Statistical significance was defined as p < 0.05.

#### Heterogeneity

Heterogeneity was evaluated using the Q-statistic (within the design) and the I^2^ statistic, considering I^2^ of > 50% as substantial heterogeneity. Additionally, the incoherence of network models was evaluated using between-study variance, a random-effects design-by-treatment interaction model, and node-splitting analysis [32].

#### Publication bias

Finally, publication bias was evaluated using a funnel plot when the number of studies exceeded 10 in primary outcomes, with asymmetry indicating the potential presence of publication bias with the Egger’s asymmetry test for further confirmation [30].

## Results

### Study selection

**Fig. 1:**
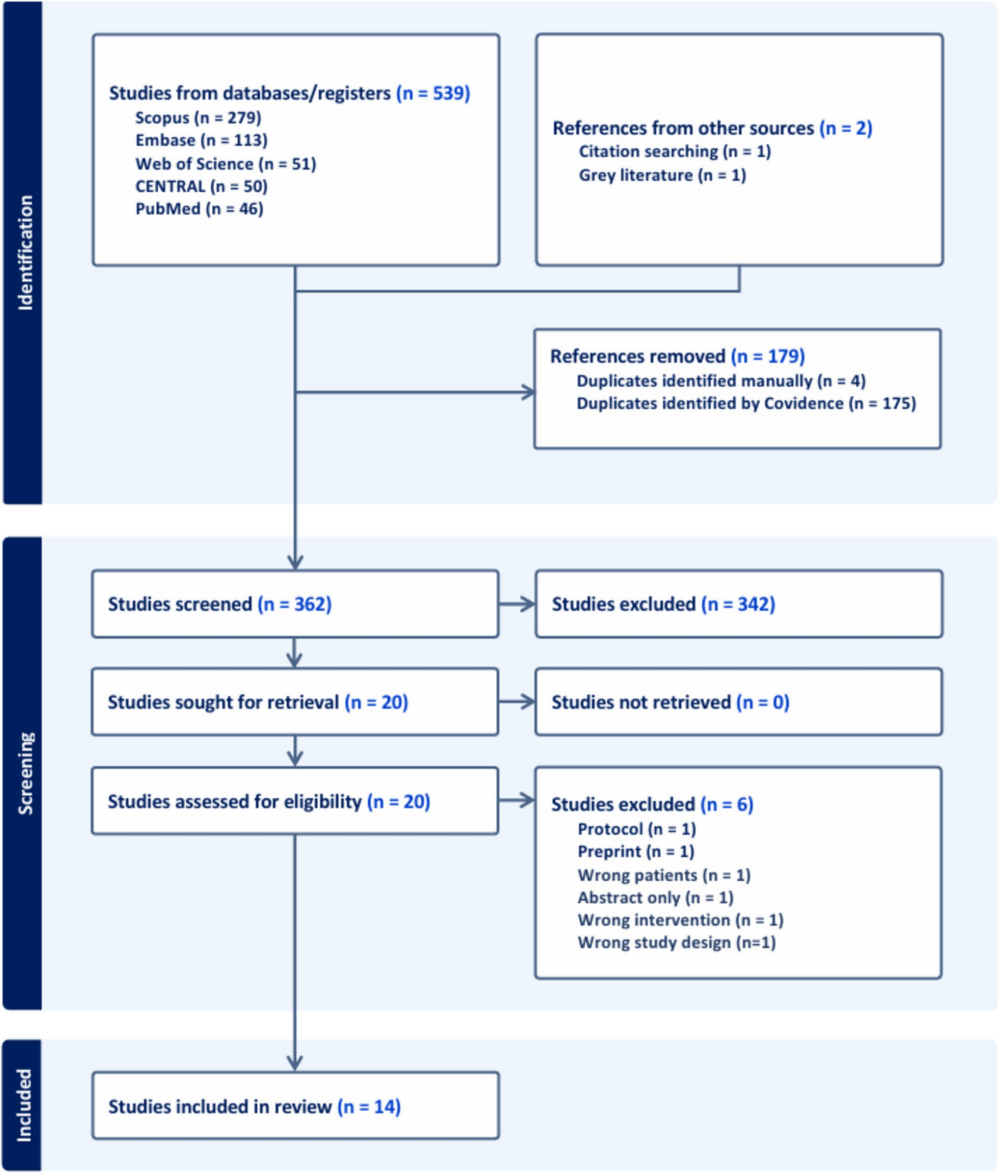
PRISMA flow chart for the systematic search and selection process

The systematic search identified 539 records from databases and two additional records from other sources. Following removing 179, including duplicates by Covidence software, 362 unique records were screened by title and abstract. Of these, 342 were excluded for not meeting the inclusion criteria. The remaining 20 studies underwent full-text review, with six subsequently excluded (Supplementary Table 3). Ultimately, 14 trials [14–27] met the eligibility criteria and were included in the final analysis. The PRISMA flow diagram summarizes the study selection process **(**Fig. 1**)**.

### Characteristics of included studies

Fourteen RCTs with 4,764 participants were included. All studies were conducted in China and involved adult patients scheduled for colonoscopy. Among the included trials, five exclusively enrolled patients with functional or chronic constipation, five did not exclude patients with constipation, and four specifically excluded them. The intervention regimens varied, combining PEG with Lin in different doses. Two trials evaluated both low- and high-volume PEG regimens, four exclusively used high-volume PEG (3–4 L), and the remaining trials utilized low-volume regimens (2 L), with one trial (Zhang et al., 2023) employing an ultra-low-volume regimen (1L PEG + 2Lin). Control groups uniformly used PEG-only preparations, predominantly with 3L or 4L PEG. A detailed summary of the characteristics of the included trials is shown in (Table 1).

**Table 1:** Summary overview of the included trials

Study ID	Study Design	Country	Sample Size, n	Study Arms^*^			Patient Criteria			Primary Outcome (s)
				**Intervention (s)**		**Control (s)**	**Age, y**	**Status**	**Constipation**	
				**Combination (s)**	**Volume**^**#**^					
**Song et al. 2024**	Single-blinded, RCT	China	260	3L-PEG + 6 Lin and 2L-PEG + 6 Lin	Low & High	4L-PEG and 3L-PEG	18 to 70	Scheduled for colonoscopy	With chronic constipation	BBPS & OBPS
**Liu et al. 2024**	Single-blinded, RCT	China	753	2L-PEG + 1 Lin and 2L PEG + 2 Lin	Low	3L-PEG	18 to 65	Scheduled for a colonoscopy	Not excluded (18.6% with chronic constipation)	OBPS
**Wang et al. 2024**	Single-blinded, RCT	China	502	3L-PEG + 1 Lin, 3L-PEG + 3 Lin, and 4L-PEG + 1 Lin	High	4L-PEG	≥ 18	Willingly underwent colonoscopy	With functional constipation	BBPS
**Xu et al. 2024**	Single-blinded, RCT	China	322	3L-PEG + 3 Lin	High	4L-PEG	18 to 75	Scheduled to undergo diagnostic, screening, or surveillance colonoscopy	With constipation	BBPS & Adequate preparation
**Xinlei et al. 2024**	Open-label, RCT	China	612	2L-PEG + 1 Lin	Low	3L-PEG	18 to 70	Indications for colonoscopy	Excluded (0%)	OBPS
**Tongchang et al. 2023**	Single-blinded, RCT	China	130	2L-PEG + 3 Lin and 3L-PEG + 3 Lin	Low & High	3L-PEG	18 to 75	Intended to undergo diagnostic, screening, or surveillance colonoscopy	Not excluded	Adequate preparation
**Qiliang et al. 2023**	Double-blinded, RCT	China	75	2L-PEG + 1 Lin	Low	2L-PEG and 3L-PEG	> 18	Scheduled for a colonoscopy	Not excluded	BBPS
**Wu et al. 2023**	Double-blinded, RCT	China	120	3L-PEG + 3 Lin	High	3L-PEG	18 to 70	Scheduled for a colonoscopy	With functional constipation	BBPS
**Yang et al. 2023**	Single-blinded, RCT	China	266	2L PEG + 2 Lin	Low	4L-PEG and 2L-PEG	18 to 80	Scheduled for a colonoscopy	Excluded (0%)	BBPS
**Xiaxi et al. 2023**	Single-blinded, RCT	China	310	2L PEG + 2 Lin	Low	3L-PEG	18 to 75	Willing and able to tolerate colonoscopy	Excluded (0%)	BBPS
**Zhang et al. 2023**	Double-blinded, RCT	China	568	1L-PEG + 2 Lin	Ultra-Low	2L-PEG	18 to 70	Low-risk patients scheduled for colonoscopy	Excluded (0%)	BBPS
**Tao et al. 2022**	Double-blinded, RCT	China	240	2L-PEG + 1 Lin	Low	3L-PEG	18 to 70	Undergoing ordinary or painless colonoscopy	Not excluded (4% with constipation)	BBPS
**Zhenguang et al. 2022**	Single-blinded, RCT	China	174	3L-PEG + 3 Lin	High	3L-PEG and 4L-PEG	18 to 80	Scheduled for a colonoscopy	With chronic constipation	BBPS
**Zhang et al. 2021**	Double-blinded, RCT	China	432	2L-PEG + 1 Lin	Low	2L-PEG and 4L-PEG	18 to 70	Scheduled for a colonoscopy	Not excluded (24% with constipation)	BBPS

*N* number, *Y* year, *RCT* randomized controlled trial, *L* liter, *PEG* polyethylene glycol, *Lin* Linaclotide, *BBPS* boston bowel preparation scale, *OBPS* boston bowel preparation scale

*Intervention arm: combination of number of pills of Lin and number of liters of PEG, Control arm number of liters of PEG alone, #PEG volume; High-Volume: using 3–4 L of PEG solution. Low-Volume: using 2 L of PEG solution. Ultra-Low-Volume using less than 2 L of solution (1 L PEG)

The number of patients assessed in the intervention group was 2,609, and 2,081 in the control groups. The mean age across our included trials was 51.1 years (± 11.7). The study population was nearly balanced in gender distribution, with 47.14% males and 52.86% females. A detailed summary of the included patients’ characteristics is shown in Table 2.

**Table 2:** Baseline characteristics of the included patients

Study ID	Groups*	Total, n	Age, y, m (SD)	Sex, male, n (%)	BMI, m (SD)	Constipation, n (%)
**Song et al. 2024**	**2L-PEG + 6 Lin**	63	±12.2 51.6	20 (34.92)	22.8 ±3.6	63 (100)
	**3L-PEG + 6 Lin**	65	49.2 ±12.6	23 (35.38)	23 ±2.5	65 (100)
	**3L-PEG**	63	49.8 ±12.4	27 (42.86)	23.2 ±2.6	63 (100)
	**4L-PEG**	64	49.7 ±9.4	25 (39.05)	23 ±3.1	64 (100)
**Liu et al. 2024**	**2L-PEG + 1 Lin**	248	51.54 ±9.9	115 (46.4)	23.1 ±3.2	40 (16.1)
	**2L PEG + 2 Lin**	250	50.14 ±11.6	126 (50.4)	23.33 ±3.1	44 (17.6)
	**3L-PEG**	251	50.7 ±12	127 (50.6)	23.65 ±3.5	56 (22.3)
**Wang et al. 2024**	**3L-PEG + 1 Lin**	120	51.22 ±6.5	20 (15.6)	23.1 ±2.7	120 (100)
	**3L-PEG + 3 Lin**	128	49 ±10	28 (21.9)	22.3 ±3.15	128 (100)
	**4L-PEG + 1 Lin**	128	48.4 ±11.6	28 (23.3)	23.12 ±3.25	128 (100)
	**4L-PEG**	126	50 ±10.4	22 (17.5)	23.5 ±3.46	126 (100)
**Xu et al. 2024**	**3L-PEG + 3 Lin**	160	53.7 ±11.9	82 (51.2)	22.9 ±2.1	160 (100)
	**4L-PEG**	159	53.2 ±11.5	85 (53.5)	23.06 ±1.9	159 (100)
**Xinlei et al. 2024**	**2L-PEG + 1 Lin**	399	54 ±9	222 (55.6)	25 ±3.36	0 (0)
	**4L-PEG**	202	53 ±9	98 (48.5)	24.5 ±11.5	0 (0)
**Tongchang et al. 2023**	**2L-PEG + 3 Lin**	41	**≥50: **26 (63%), **<50:** 15 (37%)	23 (56.1)	22.5 ±2.6	NM
	**3L-PEG + 3 Lin**	46	**≥50: **26 (56%), **<50:** 20 (44%)	22 (47.8)	22.6 ±2.8	NM
	**3L-PEG**	43	**≥50:** 19 (44%), **<50:** 24 (56%)	23 (53.5)	22.9 ±3.3	NM
**Qiliang et al. 2023**	**2L-PEG + 1 Lin**	25	67.3 ±5.5	13 (52)	NM	NM
	**2L-PEG**	25	65.76 ±6	13 (52)	NM	NM
	**3L-PEG**	25	67.4 ±5.85	14 (56)	NM	NM
**Wu et al. 2023**	**3L-PEG + 3 Lin**	50	48.9 ±9.8	24 (48)	NM	50 (100)
	**3L-PEG**	50	48.3 ±10.3	23 (46)	NM	50 (100)
**Xiaxi et al. 2023**	**2L PEG + 2 Lin**	152	40.7 ±10.5	79 (51.3)	NM	0 (0)
	**3L-PEG**	152	39.33 ±12	75 (48.7)	NM	0 (0)
**Yang et al. 2023**	**2L PEG + 2 Lin**	142	48.6 ±13.3	67 (47.18)	NM	0 (0)
	**2L-PEG**	12	50.8 ±16	4 (33.33)	NM	0 (0)
	**4L-PEG**	112	49.66 ±12.1	50 (44.6)	NM	0 (0)
**Zhang et al. 2023**	**1L-PEG + 2 Lin**	273	45.1 ±11.8	140 (51.3)	22.43 ±2.11	0 (0)
	**2L-PEG**	275	46.36 ±11.5	156 (56.7)	22.5 ±2.13	0 (0)
**Tao et al. 2022**	**2L-PEG + 1 Lin**	117	52.4 ±7.3	63 (53.8)	NM	10 (4)
	**3L-PEG**	118	53.9 ±8.4	66 (55.9)	NM	
**Zhenguang et al. 2022**	**3L-PEG + 3 Lin**	58	58.1 ±13.26	27 (46.55)	NM	58 (100)
	**3L-PEG**	58	57.7 ±13.8	32 (55.2)	NM	58 (100)
	**4L-PEG**	58	61.3 ±10	30 (51.7)	NM	58 (100)
**Zhang et al. 2021**	**2L-PEG + 1 Lin**	144	50.7 ±10.9	74 (51.4)	24.3 ±3.9	34 (23.6)
	**2L-PEG**	144	50.2 ±10.9	70 (55.6)	24.3 ±3.2	31 (21.5)
	**4L-PEG**	144	52.2 ±10.4	75 (52.1)	24.6 ±3.7	39 (27)

Data are presented in mean ±SD or proportions as (%)

*N* number, *M* mean, *Y* year, *L* liter, *PEG* polyethylene glycol, *Lin* linaclotide, *NM* not mentioned

*Intervention arm combination of number of pills of Lin and number of liters of PEG, Control arm number of liters of PEG alones

### Quality assessment

By RoB2 [31], we found that while most studies demonstrated a low overall risk (n = 9), five RCTs showed some risks: three studies [16, 19, 22] were rated as high risk, and two [17, 26] had some concerns. The primary concerns in these five studies were concentrated in domains D1 (bias in the randomization process) and D4 (bias in the measurement of the outcomes), as assessed using the RoB2 tool. These issues were mainly attributed to inadequate reporting of the randomization process and the unblinded assessment of the subjective primary outcomes. The remaining domains were consistently rated as low risk across the included studies. The RoB diagram summarizes the assessment in (Supplementary Fig. 1).

### Primary outcomes

#### BBPS score & preparation success

**Fig. 2:**
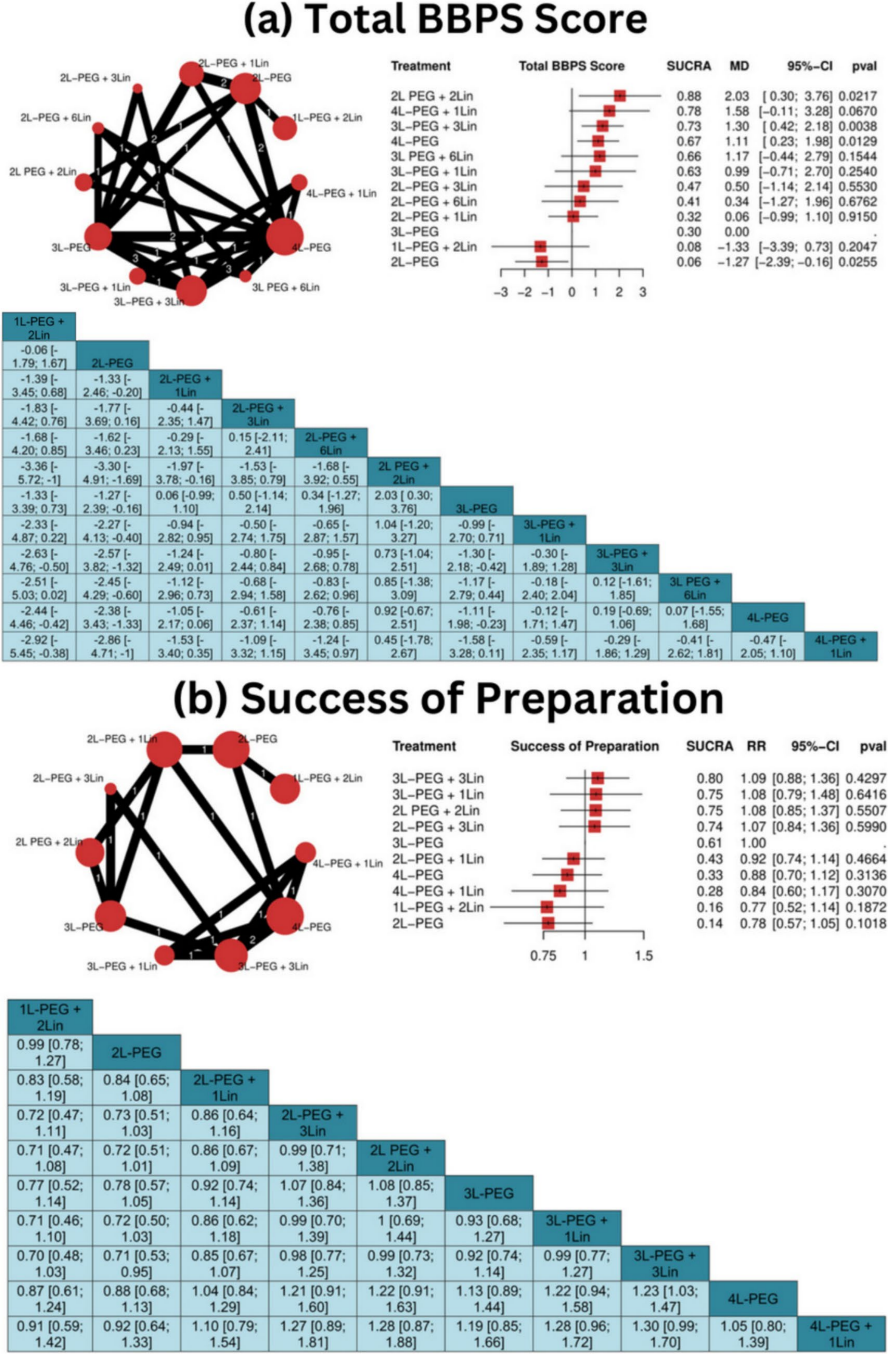
Network geometry, forest plot, and league tables for total BBPS score and preparation success. *BBPS* Boston Bowel Preparation Scale, *L* liter, *Lin* linaclotide, *PEG* polyethylene glycol, *SUCRA* Surface Under the Cumulative Ranking Area, *RR* risk ratio, *CI* confidence interval

**Fig. 3:**
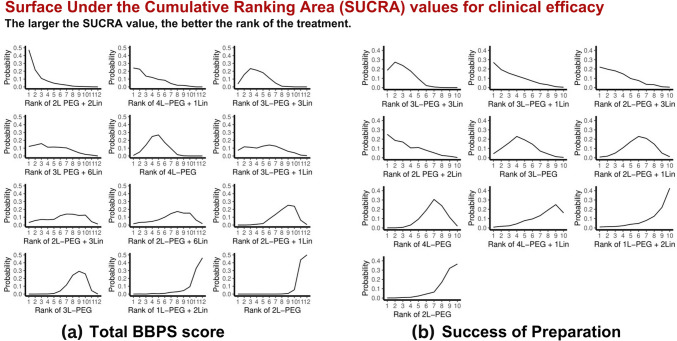
Surface Under the Cumulative Ranking Area (SUCRA) for total BBPS score and preparation success. *BBPS* Boston Bowel Preparation Scale, *L* liter, *Lin* linaclotide, *PEG* polyethylene glycol

The analysis of BBPS scores across different volume of PEG with different number of pills of Lin (11 studies, 3,024 participants) showed significant improvements with 2L PEG + 2Lin (MD = 2.03, 95% CI: 0.30 to 3.76, *P* = 0.0217, SUCRA = 0.88), 3L-PEG + 3Lin (MD = 1.30, 95% CI: 0.42 to 2.18, *P* = 0.0038, SUCRA = 0.73), and 4L-PEG (MD = 1.11, 95% CI: 0.23 to 1.98, *P* = 0.0129, SUCRA = 0.67) compared to 3L-PEG **(**Fig. 2a and 3a**)**. However, heterogeneity was very high (I^2^ = 96%, *P* < 0.01).

Most comparisons did not show evidence of incoherence, except for 2L-PEG vs. 2L PEG + 2Lin (*P* = 0.0029), 2L-PEG vs. 2L-PEG + 1Lin (*P* = 0.0274), and 2L PEG + 2Lin vs. 4L-PEG (*P* = 0.0029), which suggests inconsistency between the direct and indirect estimates (Supplementary Table 4).

The analysis of preparation success rate included six studies (2,668 participants) comparing various bowel preparation regimens to the 3L-PEG regimen. No statistically significant differences were observed for any comparison **(**Fig. 2b and 3b**)**. Heterogeneity was high (I^2^ = 83.5% *P* = 0.0023). No evidence of incoherence was found between all the interventions, suggesting that the direct and indirect estimates were consistent in all the comparisons (Supplementary Table 5).

#### Publication bias

In the comparison-adjusted funnel plots, symmetry was observed across all primary outcomes. Egger's regression test revealed no significant evidence of publication bias, including for the total BBPS score (*P* = 0.4147) and preparation success (*P* = 0.7079) (Supplementary Fig. 2 and 3).

### Secondary outcomes

#### Efficacy outcomes

For total OBPS scores (three studies, 1,605 participants), significant reductions compared to 3L-PEG were noted with 2L PEG + 2Lin (MD = −0.62, 95% CI: −0.99 to −0.24, *P* < 0.01), 3L-PEG + 6Lin (MD = −1.37, 95% CI: −2.10 to −0.64, *P* < 0.01), and 4L-PEG (MD = −1.25, 95% CI: −1.98 to −0.52, *P* = 0.01) (Supplementary Fig. 4a). These findings suggest that these regimens reduce overall burden scores. Heterogeneity was not significant (I^2^ = 45.9%, *P* = 0.1739), providing relatively consistent results.

For fluid volume (3 studies, 1,605 participants), only 2L-PEG + 6Lin significantly reduced fluid volume compared to 3L-PEG (MD = −0.24, 95% CI: −0.42 to −0.06, *P* = 0.0102) and other regimens showed no significant differences (Supplementary Fig. 4b). Heterogeneity was not significant (I^2^ = 43.9%, *P* = 0.1820).

**Fig. 4:**
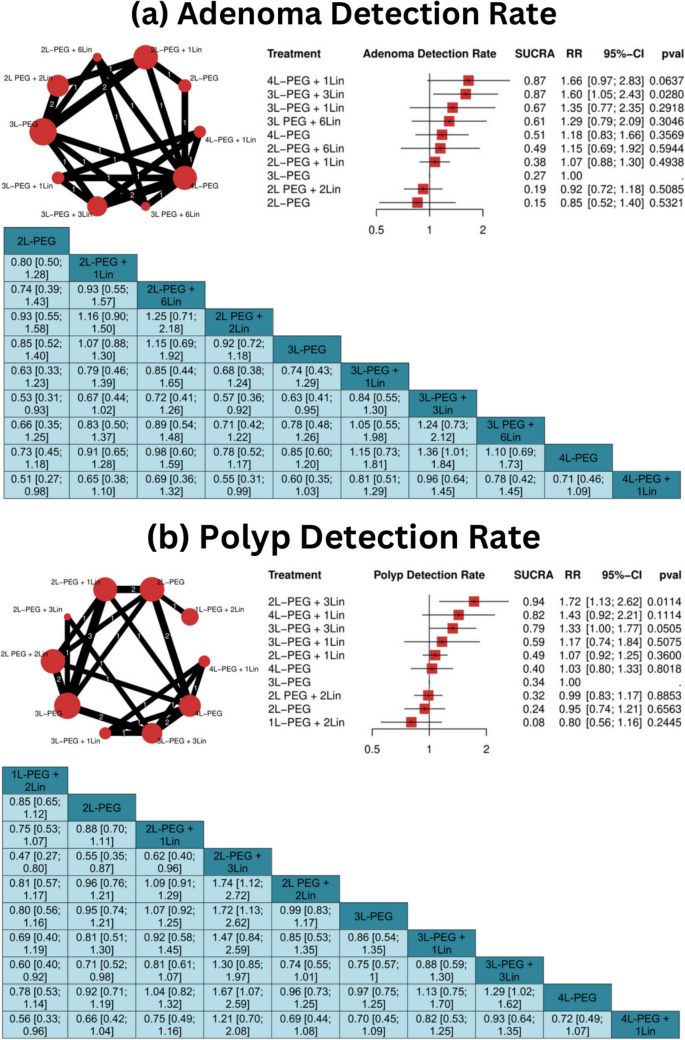
Network geometry, forest plot, and league tables for adenoma and polyp detection rates. *L* liter, *Lin* linaclotide, *PEG* polyethylene glycol, *SUCRA* Surface Under the Cumulative Ranking Area, *RR* risk ratio, *CI* confidence interval

In the analysis of ADR (8 studies, 2,872 participants), 3L-PEG + 3Lin significantly improved detection rates (RR = 1.60, 95% CI: 1.05 to 2.43, *P* = 0.0280) **(**Fig. 4a**)**. Heterogeneity was negligible (I^2^ = 0%, *P* = 0.6731), supporting consistency in these findings.

For PDR (11 studies, 3,636 participants), significant improvements were observed with 2L-PEG + 3Lin (RR = 1.72, 95% CI: 1.13 to 2.62, *P* = 0.0114) and 3L-PEG + 3Lin (RR = 1.33, 95% CI: 1.00 to 1.77, *P* = 0.0505) **(**Fig. 4b**)**. Heterogeneity was low (I^2^ = 24.4%, *P* = 0.2188), indicating consistent results across studies.

For procedure times, significant reductions were seen with 3L-PEG + 3Lin (insertion time: MD = −1.28, 95% CI: −2.14 to −0.42, *P* < 0.01; withdrawal time: MD = −1.56, 95% CI: −2.69 to −0.43, *P* < 0.01) (Supplementary Fig. 5b and 6). Total procedure time decreased significantly with 3L-PEG + 1Lin (MD = −2.29, 95% CI: −4.42 to −0.16, *P* = 0.035), 3L-PEG + 3Lin (MD = −4.76, 95% CI: −6.24 to −3.27, *P* < 0.01), and 4L-PEG (MD = −3.12, 95% CI: −4.61 to −1.64, *P* < 0.01) (Supplementary Fig. 5a). Moderate heterogeneity (I^2^ = 64.7%, *P* = 0.058) was observed, suggesting these effects are relatively stable across studies.

The time to first defecation (6 studies, 3,017 participants) was significantly shorter with 2L-PEG + 1Lin (MD = −20.02, 95% CI: −35.21 to −4.84, *P* = 0.0098) (Supplementary Fig. 7). Heterogeneity was high (I^2^ = 92.4%, *P* < 0.0001), indicating substantial variability in study outcomes.

#### Safety outcomes

**Fig. 5:**
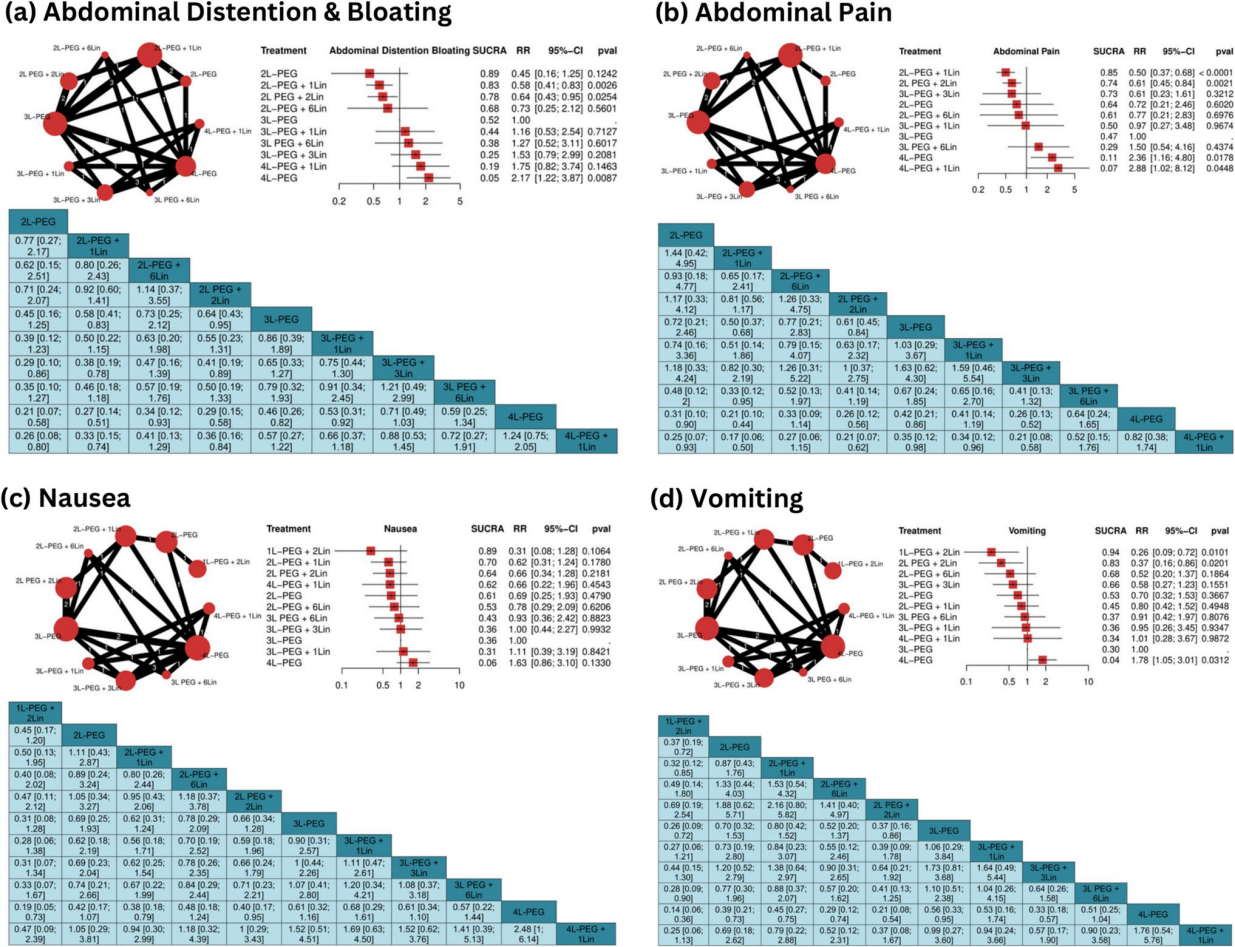
Network geometry, forest plot, and league tables for adverse events Including: **a** Abdominal distention & bloating, **b** Abdominal pain, **c** Nausea, and **d** Vomiting. *L* liter, *Lin* linaclotide, *PEG* polyethylene glycol, *SUCRA* Surface Under the Cumulative Ranking Area, *RR* risk ratio, *CI* confidence interval

The analysis of the safety data from the included studies showed for abdominal pain, reduced risks with 2L-PEG + 1Lin (RR = 0.50, 95% CI: 0.37 to 0.68, *P* < 0.01) and 2L PEG + 2 Lin (RR = 0.61, 95% CI: 0.45 to 0.84, *P* = 0.021). Conversely, increased risks were observed with 4L-PEG (RR = 2.36, 95% CI: 1.16 to 4.80, *P* = 0.0178) and 4L-PEG + 1Lin (RR = 2.88, 95% CI: 1.02 to 8.12, *P* = 0.0448) **(**Fig. 5b**)**, with low heterogeneity (I^2^ = 0%, *P* = 0.4265).

For abdominal distension/bloating (9 studies, 3,399 participants), significant reductions were observed with 2L-PEG + 1Lin (RR = 0.58, 95% CI: 0.41 to 0.83, *P* = 0.0026) and 2L PEG + 2Lin (RR = 0.64, 95% CI: 0.43 to 0.95, *P* = 0.0254). Conversely, 4L-PEG increased the risk (RR = 2.17, 95% CI: 1.22 to 3.87, p = 0.0087) **(**Fig. 5a**)**, with moderate heterogeneity (I^2^ = 36.1%, *P* = 0.1295).

No increased risk for nausea (8 studies, 3,271 participants) compared with 3L-PEG **(**Fig. 5c**)**. Heterogeneity was high (I^2^ = 72.8%, *P* = 0.0025), reflecting variability in study outcomes and inconsistency between designs, limiting the generalizability of results.

For vomiting, 1L-PEG + 2Lin (RR = 0.26, 95% CI: 0.09 to 0.72, *P* = 0.01) and 2L PEG + 2Lin (RR = 0.37, 95% CI: 0.16 to 0.86, *P* = 0.0201) reduced the risk, while 4L-PEG increased it (RR = 1.78, 95% CI: 1.05 to 3.01, *P* = 0.0312) **(**Fig. 5d**)**. Minimal heterogeneity (I^2^ = 0%, *P* = 0.5904) supports the reliability of these findings.

For combined nausea and vomiting (4 studies, 1,042 participants), a significant reduction was observed with 2L-PEG + 1Lin (RR = 0.50, 95% CI: 0.31 to 0.79, *P* = 0.0030) (Supplementary Fig. 8). No heterogeneity was reported (I^2^ = 0%, *P* = 0.7753), indicating consistency in results.

For poor sleep quality, only 2L-PEG + 1Lin (RR = 0.62, 95% CI: 0.44 to 0.89, P = 0.0087) reduce the risk compared to 3L-PEG, with no significant differences in all other regimens (Supplementary Fig. 9a). No heterogeneity was reported (I^2^ = 37%, P = 0.1561), indicating consistency in results.

#### Compliance outcome

Across the included studies, willingness to repeat bowel preparation was commonly evaluated as a secondary outcome using patient questionnaires administered either before or after colonoscopy. Most assessments involved a simple binary response (“yes” or “no”), often documented by nurses or research staff, with others did not state the tool for assessment.

For willingness to repeat preparation, no statistically significant differences were observed between regimens compared to 3L-PEG (Supplementary Fig. 9b). High heterogeneity (I^2^ = 81.2%, *P* < 0.01).

## Discussion

Our network meta-analysis evaluated the efficacy and safety of various bowel preparation regimens combining different volumes of PEG with Lin, using 3L-PEG as the central comparator. Bowel cleanliness, assessed by the BBPS, improved significantly with 2L PEG + 2Lin, 3L-PEG + 3Lin, and 4L-PEG. Adenoma detection was highest with 3L-PEG + 3Lin, while polyp detection improved with 2L PEG + 3Lin. Procedure times were notably reduced with 3L-PEG + 3Lin. Vomiting risks were lower with 1L PEG + 2Lin, and abdominal pain and bloating decreased with 2L PEG + 1Lin and 2L PEG + 2Lin but increased with 4L-PEG. Safety outcomes demonstrated low heterogeneity, supporting the reliability of these results.

Effective bowel preparation with PEG is crucial for successful colonoscopy outcomes, as it enhances mucosal visualization and optimizes conditions for polypectomy techniques, which demonstrate high efficacy and safety in polyp removal [33, 34]. Since its introduction in 1980, PEG has remained the first-line bowel cleansing regimen in most clinical endoscopy centers. While various laxatives are available, studies evaluating Lin as a bowel cleanser remain limited [35]. Studies of Lin as a bowel cleanser are currently very limited. A meta-analysis of 31 studies involving over 370,000 colonoscopies found that inadequate bowel preparation had a greater impact on detecting early colon lesions than advanced lesions [36]. This underscores the importance of proper bowel cleansing in improving diagnostic accuracy and clinical outcomes.

ADR is a key indicator of colonoscopy quality, with evidence showing that a 1% increase in ADR reduces colorectal cancer incidence by 3% and mortality by 5% [37]. Despite its clinical significance, some studies have found no significant differences in polyp or adenoma detection across different bowel preparation regimens, possibly due to insufficient evidence or study limitations. However, several studies have explored the impact of PEG combined with Lin on bowel preparation quality and ADR. Zhang et al. (2021) found that 2L PEG + Lin resulted in a significantly higher percentage of adequate bowel preparation than 2L PEG alone (87.9% vs. 77.0%) but was comparable to 4L PEG (87.9% vs. 91.4%) [18]. Wang et al. (2024) conducted a randomized, multicenter trial across seven hospitals, where patients with chronic constipation undergoing colonoscopy were randomized into four split-dose PEG regimens: 4L-PEG alone, 4L PEG + 1-day Lin, 3L-PEG + 1-day Lin, and 3L-PEG + 3-day Lin. The study assessed bowel preparation adequacy (BBPS ≥ 6), ADR, PDR, AEs, sleep quality, and willingness to repeat the procedure. However, no significant differences in adenoma or polyp detection rates were observed across the groups [20].

Similarly, Yang et al. (2023) reported that compound PEG electrolyte powder combined with Lin provided bowel cleansing quality comparable to 4L PEG, with fewer AEs, and was superior to 2L PEG alone [38]. Liu et al. (2024) conducted a randomized trial with 753 patients, comparing 2L PEG + 2Lin, 2L PEG + Lin, and 3L PEG. The 2L PEG + 2Lin group had the highest success rate of bowel preparation (92.0%), significantly outperforming both 2L PEG + Lin (82.3%) and 3L PEG (82.1%). While 2L PEG + Lin and 3L PEG had comparable efficacy, the 2L PEG + 2Lin group achieved superior cleansing in the right and mid-colon, particularly in patients with chronic constipation. Additionally, adverse reactions were comparable between the 2L PEG + 2Lin and 2L PEG + Lin groups but were superior to the 3L PEG group [15].

In our study, adenoma detection was highest with 3L-PEG + 3Lin, while polyp detection was most improved with 2L PE + 3Lin. Our findings support the potential role of PEG + Lin regimens in improving bowel preparation, though further research is required to assess their direct impact on ADR and PDR. A study comparing various bowel preparation regimens found that 3L PEG + Lin achieved superior bowel cleanliness, as assessed by BBPS, compared to both 3L PEG and 2L PEG + Lin, while demonstrating comparable efficacy to 4L PEG [14]. Additionally, a multicenter randomized controlled trial reported that 2L PEG combined with Lin resulted in significantly higher overall bowel cleansing scores compared to 3L PEG. Patients in the 2L PEG + Lin group also experienced lower rates of bloating, discomfort, and intolerability before colonoscopy [38]. Our meta-analysis further corroborates these findings, demonstrating significant improvements in BBPS scores with 2L PEG + 2Lin, 3L PEG + 3Lin, and 4L PEG. Notably, adenoma detection was highest with 3L PEG + 3Lin, while polyp detection was enhanced with 2L PEG + 3Lin.

Several studies have investigated the incidence of vomiting and abdominal pain associated with various bowel preparation regimens, particularly focusing on combinations PEG with Lin. A randomized, controlled, multicenter trial evaluated the efficacy and safety of various split-dose PEG regimens combined with Lin in patients with chronic constipation undergoing colonoscopy [20]. The study found that 3L-PEG + 3d-Lin and 4L-PEG + 1d-Lin significantly improved bowel cleansing efficacy, as reflected in higher total BBPS scores compared to 4L-PEG alone (7.03 ± 1.24 vs. 6.00 ± 1.61 and 6.90 ± 1.28 vs. 6.00 ± 1.61 respectively). Additionally, both regimens demonstrated higher adequate bowel preparation rates (84.4% and 80.0% vs. 60.3%). However, there were no significant differences in adenoma or polyp detection rates among the groups. While the study did not specifically evaluate procedure times, it reported a lower incidence of mild AEs, including vomiting and abdominal discomfort, in the 4L-PEG + 1d-Lin group (66.7% vs. 81.7%) compared to 4L-PEG alone. A similar trend was observed with 3L-PEG + 3d-Lin (75.0% vs. 81.7%), though the difference was not statistically significant. No significant differences were noted in overall safety outcomes. These findings support the efficacy of PEG + Lin regimens in improving bowel cleansing (higher BBPS scores) while maintaining comparable safety and tolerability to 4L-PEG alone. This aligns with the network meta-analysis, which demonstrated BBPS improvements and better tolerability with PEG + Lin combinations. [20].

Sulz et al. analyzed numerous colonoscopies and revealed that inadequate preparation significantly impairs early lesion detection [36]. Despite the availability of various laxatives introduced decades ago, the PEG remains the first-line choice in most endoscopy centers due to its proven efficacy. However, its high volume and unpleasant taste remain notable drawbacks [7, 35]. Various studies have explored strategies to reduce PEG's side effects. Combining PEG with lubiprostone improves adequate bowel preparation rates without altering BBPS scores [39]. Similarly, pairing PEG with mosapride enhances BBPS scores and preparation efficacy while reducing AEs, though it has a limited impact on achieving adequate preparation rates [40].

In our study, bowel cleanliness, measured by BBPS scores, improved significantly with Lin-enhanced regimens. Specifically, 2L PEG + 2 Lin, 3L-PEG + 3Lin, and 4L-PEG achieved better preparation quality than 3L-PEG alone, with comparable overall success rates. Yang et al. also found that 2L-PEG combined with two capsules of Lin (2LPEG + 2Lin) achieved significantly higher BBPS scores across all colonic segments compared to 2L-PEG alone and matched the performance of 4L-PEG while showing fewer adverse reactions and better patient tolerability [26]. Xu et al. demonstrated that 3L-PEG combined with Lin (3L-PEG + 3Lin) achieved significantly higher rates of adequate bowel preparation than 4L-PEG, with enhanced right colon cleansing likely due to Lin's ability to promote intestinal motility [21].

Interestingly, our analysis found that the 2L PEG + 2Lin regimen led to greater BBPS improvements than the more intensive 3L PEG + 3Lin. While seemingly counterintuitive, several factors may explain this result. Studies using 2L PEG + 2Lin often included lower-risk patients, such as those without chronic constipation, potentially biasing outcomes. Lower-volume regimens tend to be better tolerated, improving compliance and completion rates. In contrast, higher volumes may reduce adherence due to discomfort [7, 10]. A ceiling effect may also contribute—beyond a certain point, increasing PEG volume offers limited additional cleansing benefits, especially when combined with an effective adjunct such as Lin. These factors underscore the interplay between volume, patient selection, and tolerability in bowel preparation efficacy.

Also, in the study by Wang et al., 4L-PEG + 1Lin significantly outperformed 4L-PEG in achieving higher BBPS scores and adequate bowel preparation rates. Similarly, 3L-PEG + 3Lin surpassed 3L-PEG + 1Lin in both metrics. The authors hypothesized that Lin, acting as a guanylate cyclase-C receptor agonist, and PEG, functioning as an osmotic laxative, enhance bowel cleanliness through complementary mechanisms [14]. Collectively, these findings emphasize Lin's utility as an adjunct to PEG regimens, improving bowel preparation quality while reducing AEs and enhancing patient compliance.

The BBPS is a continuous measure that detects modest, segment-specific improvements in cleansing, whereas “adequate bowel preparation” is a binary endpoint that registers only whether a pre-set threshold has been achieved (typically total BBPS ≥ 6 with ≥ 2 in every segment). [41–43] In the six studies that reported “adequate preparation,” control‐group BBPS values already clustered near this threshold (mean ≈ 5.9–6.3). With such a ceiling effect, 2L PEG + 2 Lin produced a statistically significant rise in mean BBPS but did not shift enough additional patients from “inadequate” to “adequate” to influence the dichotomous outcome. In practical terms, the regimen improved overall mucosal visibility, yet the magnitude of improvement was insufficient to change the proportion of examinations meeting the definition of adequacy.

Our study demonstrated that adding Lin to PEG regimens improved adenoma and polyp detection rates. Adenoma detection was highest with 3L-PEG + 3Lin, while polyp detection was notably enhanced with both 2L-PEG + 3Lin and 3L-PEG + 3Lin. These findings suggest that Lin contributes to improved bowel cleanliness and enhances diagnostic outcomes. Xu et al. supported these findings, showing that 3L-PEG + Lin offered better tolerability and fewer side effects like nausea, bloating, and abdominal discomfort compared to 4L-PEG, with improved patient satisfaction due to the lower volume and reduced AEs [21]. Similarly, Tongchang et al. reported that 2L-PEG + Lin had higher completion rates and patient willingness to repeat the regimen compared to other regimens, underscoring its acceptability and effectiveness for bowel preparation [23].

Adenoma detection is influenced not only by the cleansing quality but also by bowel distension, peristalsis, patient comfort, and withdrawal technique. The significant increase in ADR observed with the 3L-PEG + 3Lin regimen likely reflects enhanced proximal colon cleansing due to Lin’s promotility and secretory effects, especially in the right colon—where flat lesions are often missed. In addition, improved patient tolerability and reduced bloating may lead to more thorough withdrawal and inspection time. Since a 1% increase in ADR corresponds to a 3% reduction in colorectal cancer incidence and a 5% reduction in mortality [37, 44],), combining 3L PEG with Lin may offer both procedural and long-term oncologic benefit.

Regarding procedural efficiency, our study highlighted significant reductions in procedure times with Lin-enhanced regimens, particularly 3L-PEG + 3Lin, which showed decreased insertion and withdrawal times. Other regimens, such as 3L-PEG + 1Lin and 4L-PEG, also resulted in shorter total procedure durations, indicating the efficiency of combining PEG with Lin. Although Xu et al. found no significant differences in cecal intubation or operation times between regimens [21], Wang et al. highlighted that total examination times were the shortest with 3L-PEG + 3Lin, reinforcing its procedural efficiency [20].

Regarding tolerability, our study demonstrated reduced vomiting risks with 1L-PEG + 2Lin and 2L-PEG + 2Lin, while 4L-PEG was associated with an increased risk. Combining nausea and vomiting scores showed notable reductions with 2L-PEG + 1Lin. Yang et al. reported that 2L-PEG + 2Lin significantly reduced bloating, abdominal pain, nausea, and vomiting compared to 4L-PEG while maintaining bowel cleanliness and improving patient comfort and sleep quality [26]. Finally, abdominal pain and bloating were significantly reduced with 2L-PEG + 1Lin and 2L-PEG + 2Lin, whereas higher volumes, such as 4L-PEG or 4L-PEG + Lin, increased these symptoms. Yang et al. reported similar findings, highlighting 2L-PEG + 2Lin's ability to maintain bowel cleanliness while reducing AEs and improving patient tolerance [26]. Zhang et al. found that 1L-PEG combined with two capsules of Lin achieved similar efficacy to 2L-PEG with no severe AEs, reducing time to first defecation, nausea, and vomiting but showing minimal impact on abdominal symptoms, likely due to the lower preparation volume [27].

AEs were reported across all included studies, with the most frequently cited symptoms being nausea, vomiting, abdominal pain, bloating or distension, and fatigue. Several studies also assessed sleep quality, noting improvements with lower-volume regimens. The number of distinct AE symptoms reported per study ranged from three to eight. Regimens combining Lin with lower PEG volumes (e.g., 2L) consistently demonstrated a lower overall incidence of AEs than high-volume PEG regimens (e.g., 4L PEG), with fewer reports of gastrointestinal discomfort and better tolerability. However, most studies listed AEs by symptoms without attributing causality to PEG or Lin. As such, these findings reflect the tolerability of the regimen as a whole rather than the isolated effect of individual components.

Generally, the efficacy of bowel preparation regimens appears to be influenced not only by the volume of PEG used but also by the number and timing of Lin doses. Lower-volume regimens (e.g., 2L PEG + 2Lin) were associated with improved BBPS scores and better tolerability, likely due to enhanced patient compliance and synergistic pharmacodynamics when Lin was administered closer to the PEG dose. In contrast, high-volume regimens like 4L-PEG, while effective in mucosal cleansing, were more frequently associated with adverse effects such as bloating and discomfort. These findings underscore the importance of tailoring preparation strategies to individual patient profiles—particularly in populations with chronic constipation or low tolerance for large-volume solutions.

## Strength

Our study has several strengths. First, it includes 14 RCTs with a large sample size of 4,764 participants, with most of the RCTs having a low risk of bias, providing robust statistical power and reliable findings. Second, it evaluates multiple PEG regimens with Lin, comprehensively comparing bowel cleansing effects, safety, and tolerability. Another point is that we analyzed each dose arm separately to identify the best combination formula of Lin and PEG. Finally, the study provides a detailed regional analysis of bowel cleanliness and incorporates both primary and secondary outcomes, offering a balanced view of efficacy and safety.

## Limitations

The study is undermined by a few limitations, including: (i) Significant heterogeneity (I^2^ > 50%) in multiple outcomes, such as BBPS scores and preparation success, limits interpretability, highlighting the variability in study protocols or populations. (ii) Reliance on published RCTs introduces potential publication bias, excluding unpublished or negative results. (iii) The studies lack long-term follow-ups, omitting sustained late safety and adherence data, reflecting that objective safety indicators like blood analyses are absent in current evidence—subjective assessments of AEs and satisfaction rate in some studies. (iv) The variability in patient populations, particularly concerning comorbidities and gastrointestinal conditions such as chronic constipation, can reduce our findings’ generalizability. (v) Another key limitation is the variability in PEG administration protocols. PEG was administered in both single and split-dose regimens within the same intervention group across different studies, and the timing of preparation initiation and Lin administration was inconsistent, affecting the comparability of results. (vi) The observed heterogeneity across BBPS and adequacy outcomes likely stems from variability in study populations (e.g., inclusion or exclusion of constipated patients) and differences in PEG dosing schedules (split vs. single). While subgroup or sensitivity analyses stratified by constipation status would be informative, such analyses are not applicable in a network meta-analysis. (vii) Notably, all the included studies were conducted in China, which may limit the generalizability of the findings to other populations with different dietary habits, healthcare systems, and genetic predispositions.

## Future directions

Future research should standardize bowel preparation protocols combining Lin with PEG for consistency across clinical settings. Longer follow-up trials, including those in other countries with diverse populations, are needed to validate findings and improve generalizability. Exploring patient-centered factors, such as satisfaction and willingness to repeat regimens using standardized methods, can optimize colonoscopy preparation. Also, key areas for further investigation include evaluating Lin-PEG regimens in special populations, such as individuals with chronic constipation and elderly patients. Tailored approaches with different PEG volumes should be assessed to determine the most effective and tolerable options, balancing bowel cleanliness, safety, and patient comfort for better clinical decision-making. Additionally, assessing the economic impact, including cost-effectiveness and healthcare resource utilization, will support evidence-based guidelines for broader clinical adoption.

## Conclusion

The current RCT-based analysis highlights the effectiveness of combining PEG with Lin compared to 3L-PEG alone. This combination may improve bowel cleanliness and detection rates while reducing AEs. Tailored regimens, such as lower-volume PEG with Lin, strike a balance between efficacy and patient comfort, with the 2L PEG + 2Lin then 3L PEG + 3Lin regimens emerging as the most effective and well-tolerated. Despite some heterogeneity, the findings overall suggest that adding Lin can enhance bowel preparation with comparable safety. Future research should aim to standardize protocols and explore novel combinations to further optimize colonoscopy preparation.

## Supplementary Information

Below is the link to the electronic supplementary material.

ESM 1 (PDF 1.51 MB)

## Data Availability

No datasets were generated or analysed during the current study.
